# HIV drug resistance and HIV transmission risk factors among newly diagnosed individuals in Southwest China

**DOI:** 10.1186/s12879-021-05854-w

**Published:** 2021-02-08

**Authors:** Xianwu Pang, Kailing Tang, Qin He, Jinghua Huang, Ningye Fang, Xinjuan Zhou, Qiuying Zhu, Xiuling Wu, Zhiyong Shen, Shujia Liang

**Affiliations:** 1 Guangxi Zhuang Autonomous Region Center for Disease Prevention and Control, Nanning, 530028 Guangxi China; 2 Guangxi Collaborative Innovation Center for Biomedicine, Guangxi Medical University, Nanning, 530021 Guangxi China

**Keywords:** Human immunodeficiency virus 1, HIV drug resistance, Primary antiretroviral resistance, Antiretroviral resistance, Transmitted drug resistance, Antiretroviral therapy

## Abstract

**Background:**

The widespread use of antiretroviral therapy (ART) has resulted in the development of transmitted drug resistance (TDR), which reduces ART efficacy. We explored TDR prevalence and its associated risk factors in newly diagnosed individuals in Guangxi.

**Methods:**

We enrolled 1324 participants who were newly diagnosed with HIV-1 and had not received ART at voluntary counselling and testing centres (VCT) in Guangxi, China, who had not received ART. Phylogenetic relationship, transmission cluster, and genotypic drug resistance analyses were performed using HIV-1 *pol* sequences. We analysed the association of demographic and virological factors with TDR.

**Results:**

In total, 1151 sequences were sequenced successfully, of which 83 (7.21%) showed evidence of TDR. Multivariate logistic regression analysis revealed that there was significant difference between the prevalence of TDR and unmarried status (adjusted odds ratio (aOR) = 2.41, 95% CI: 1.23–4.71), and CRF08_BC subtype (aOR = 2.03, 95% CI: 1.13–3.64). Most cases of TDR were related to resistance to non-nucleoside reverse transcriptase inhibitors (4.87%) and V179E was the most common mutation detected. We identified a total of 119 HIV transmission clusters (*n* = 585, 50.8%), of which 18 (15.1%) clusters showed evidence of TDR (36, 41.86%). Three clusters were identified that included drug-resistant individuals having a transmission relationship with each other. The following parameters were associated with TDR transmission risk: Unmarried status, educational level of junior high school or below, and CRF08_BC subtype may be a risk of the transmission of TDR.

**Conclusions:**

Our findings indicated that moderate TDR prevalence and highlighted the importance of continuous TDR monitoring and designing of strategies for TDR mitigation.

## Background

Guangxi is located in Southwest China, adjacent to Vietnam and the Yunnan and Guangdong provinces. It is one of the areas in China that is most heavily affected by the human immunodeficiency virus 1 (HIV-1) in China [1]. Since the identification of the first individual infected with HIV in Guangxi in 1996 [2], the number of infected individuals has increased. By the end of 2017, Guangxi has the second highest number of reported HIV cases (113,500) in the country [3]. The Free Antiretroviral Treatment Program was launched in 2003 in China, and the “Treat for All” policy was implemented in 2016, and under which HIV-positive individuals were treated after being diagnosis of HIV regardless of their CD4+ cell count [4].

The widespread use and increased coverage of ART has reduced significantly reduced the risk of HIV transmission and decreased HIV-related morbidity and mortality [5]. Meanwhile, the increase in ART access corresponds to an increase in HIV drug resistance, which can be transmitted to newly infected individuals. TDR in HIV has become a major concern as it may lead to the failure of first-line ART [6]. Certain studies have suggested that significant large variations in the prevalence of TDR can be expected in different areas worldwide based on the differences in the availability of treatments and variances in socioeconomic development [7–9]. The prevalence is relatively high in high-income countries; for e.g., it is 11.2% in the United States [10], 14.7% in Romania [11] and 9.9% in Spain [12]. In middle- and low-income countries, lower estimates of TDR the prevalence have been reported; for e.g., it is 6.3% in Latin America [8], 5.7% in India, and < 5.0% in African countries [13]. A nationwide cross-sectional survey conducted in 2015 revealed 3.6% overall prevalence of drug resistance to be in China [14]. However, more recently, the prevalence of TDR been reported to increase to 12.2% in Tianjin [15], 17.4% in Shanghai [16], and 6.12% in Beijing [17].

As the HIV epidemic continues to spread, it is essential to investigate the changing trends in HIV-1 genetics as well as the prevalence and transmission of TDR in individual locations. Here, we performed an extensive cross-sectional study on individuals newly diagnosed with HIV between 2016 and 2018 in Guangxi.

## Methods

### Study subjects and sample collection

Between January 2016 and December 2018, we enrolled 1324 individuals who were newly diagnosed (non-diagnosed earlier) with HIV-1 and had not received ART were enrolled at voluntary counselling and testing centres in Guangxi, China, who had not received ART. After obtaining the participants’ informed consent from the participants, we collected peripheral blood samples and epidemiological data. Plasma was separated within 12 h of blood collection and stored at − 80 °C until further use.

### HIV-1 RNA extraction, amplification, and sequencing

Viral RNA was extracted from the plasma samples using the QIAamp Viral RNA Mini Kit (Qiagen, Hilden, Germany) according to the manufacturer’s instructions. The target fragment of 1316 bp in the *pol* gene (HXB2: 2147–3462; encoding the protease and the first 299 residues of reverse transcriptase), which spans the reverse transcriptase and protease-encoding regions, was amplified using nested polymerase chain reaction (PCR) according to a previously described protocol [18]. PCR products of the correct size (1316 bp) were excised from the gel, purified using a gel extraction kit (Qiagen, Hilden, Germany), and sequenced on an ABI3730 sequencer (Applied Biosystems, Carlsbad, CA, USA) .

### Phylogenetic analysis

We edited all sequences with Sequencher v5.1 software (Genecodes, Ann Arbor, MI) and aligned them using BioEdit 7.1 software (Ibis Biosciences, Carlsbad, CA, USA) [17]. In order to identify the subtype of the virus gene, all the subtyping reference sequences were downloaded from the Los Alamos HIV database. The reference sequences were selected based on the following criteria: 1) inclusion of the major HIV-1 subtypes and circulating recombinant forms (CRFs); 2) covered *pol* gene sequence; 3) primarily originating from China and countries adjacent to Guangxi. In the end, 117 reference sequences were included, which covering all subtypes in China. Neighbour-joining method, which was used for the identification of gene subtypes [4, 14, 16, 17], was used to generated phylogenetic tree based on the Kimura 2-parameter model with 1000 bootstrap replicates using the MEGA7.0 software (available at: http://www.megasoftware.net) [19].

### Drug resistance analysis

We evaluated clinically relevant resistance to nucleoside reverse transcriptase inhibitors (NRTIs), non-nucleoside reverse transcriptase inhibitors (NNRTIs), or protease inhibitors (PIs) using the Stanford University HIV Drug Resistance Database Genotypic Resistance Interpretation Algorithm (version 8.8) and the International Antiviral Society Drug Resistance Mutation list [16]. The degree of drug resistance to each antiretroviral drug was categorised as susceptible, potential low-level resistance, low-level resistance, intermediate resistance, or high-level resistance.

### Transmission cluster construction

The aligned sequences were analysed using the HyPhy software to calculate the genetic distance, and the Tamura-Nei 93 pairwise genetic distance was calculated for all pairs of sequences. A genetic distance of ≤1.5% between two sequences was considered to indicate potential transmission partners [14]. The data is converted into edge lists, and the network was constructed by identifying pairs of sequences (nodes) and their potential transmission relationships (edge) using the visualisation sofware Cytoscape 3.5.1. We described the characteristics of the network, including the number of sequences (nodes), links (edges), and clusters (groups of linked sequences) [20].

### Statistical analysis

SPSS version 21.0 software (IBM, Chicago, IL, USA) was used for statistical analysis. The data in this study comprised categorical variables indicated with frequencies and percentages. Logistic regression analysis was performed to identify risk factors associated with TDR. A *P*-value < 0.05 was considered statistically significant. In case a *P*-value < 0.05 was obtained, the variable was included for further adjustment, otherwise, the variable was omitted from the adjustment.

## Results

### Distribution of HIV-1 subtypes and drug resistance

**Fig. 1:**
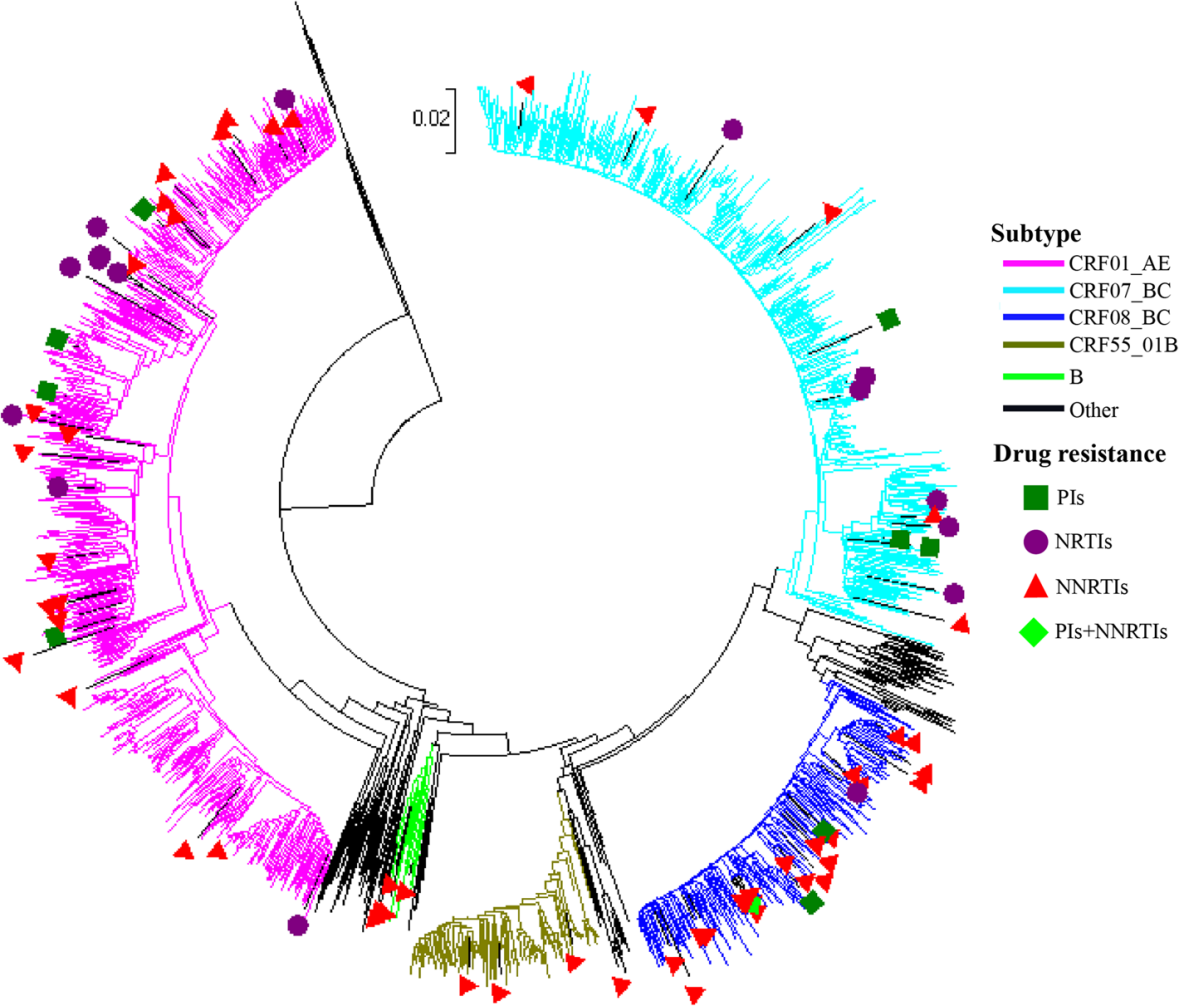
Phylogenetic tree analysis based on the sequences of human immunodeficiency virus 1 *pol* gene. We constructed a phylogenetic tree based on the human immunodeficiency virus 1 *pol* gene sequences in the plasma samples of 1151 newly diagnosed individuals. CRF, circulating recombinant form; PI, protease inhibitor; NRTI, nucleoside reverse transcriptase inhibitor; NNRTI, non-nucleoside reverse transcriptase inhibitor

In our study subjects, CRF01_AE (42.14%) was the predominant subtype, followed by CRF07_BC (30.93%), CRF08_BC (15.90%), CRF55_01B (6.86%), and others (4.17%). The three main HIV-1 subtypes accounted for 88.97% of the cases among the newly diagnosed individuals. Other subtypes included the CRFs and unique recombinant forms CRF59_01B, CRF68_01B, CRF67_01B, and 85_BC, subtype A, B, and C. The overall prevalence of drug resistance was 7.21%, including 1.56% to PIs, 1.04% to NRTIs, and 4.87% to NNRTIs (Fig. 1).

### Characteristics of the subjects and the factors associated with drug resistance

We enrolled 1324 HIV-1-infected individuals in the study, and we successfully sequenced and analysed the samples collected from 1151 (86.93%) individuals. The mean age was 40.8 years (range: 2–86 years). Most of the participants were single (46.39%), 41.62% were married, and 10.95% were divorced or widowed. Their level of educational was primarily junior high school or below (47.87%) or college (28.58%). Most of the individuals belonged to the ethnic majority Han (52.22%) and the Zhuang minority (41.96%). The major route of infection was sexual transmission; heterosexual transmission (60.21%), followed by MSM (35.19%), intravenous drug user (IDU, 4.0%) and mother-to-child transmission (MTCT, 0.6%). There was significant difference between the prevalence of TDR and marital status, subtype (all *p* values < 0.05) (Table 1).

**Table 1:** Demographic characteristics and factors associated with drug resistance (*n* = 1151)

Variables	N (%)	TDR	Crude OR (95% CI)	***p***	Adjusted OR (95% CI)^**a**^	***p***
**Age**						
< 35	508 (44.1)	26 (31.3)	1		1	
≥ 35	643 (55.9)	57 (68.7)	1.77 (1.09–2.86)	0.020	1.77 (0.9–3.52)	0.093
**Marital status**						
Married	479 (41.6)	34 (41.0)	1		1	
Unmarried	534 (46.4)	38 (45.8)	0.97 (0.6–1.58)	0.916	2.41 (1.23–4.71)	0.011
Divorced/widowed	126 (11.0)	9 (10.8)	1.01 (0.47–2.16)	0.986		
Unknown	12 (1.0)	2 (2.4)	2.62 (0.55–12.43)	0.226		
**Educational level**						
College and above	336 (29.2)	19 (22.9)	1		1	
High school or technical school	225 (19.6)	12 (14.5)	0.86 (0.4–1.84)	0.693	0.84 (0.38–1.89)	0.673
Junior high school/below	590 (51.2)	52 (62.6)	1.61 (0.94–2.78)	0.085	1.07 (0.53–2.19)	0.844
**Ethnicity**						
Han	601 (52.2)	43 (51.8)	1			
Zhuang	483 (41.9)	33 (39.8)	0.98 (0.61–1.57)	0.920		
Other	68 (5.9)	7 (8.4)	1.55 (0.67–3.61)	0.306		
**Infection route**						
MSM	405 (35.2)	18 (21.7)	1		1	
Heterosexual	693 (60.2)	62 (74.7)	0.45 (0.26–0.77)	0.004	0.61 (0.3–1.23)	0.167
Other	53 (4.6)	3 (3.6)	0.61 (0.19–2.02)	0.418		
**Sex**						
Male	919 (79.8)	63 (75.9)	1			
Female	232 (20.2)	20 (24.1)	1.3 (0.77–2.21)	0.323		
**Subtype**						
CRF01_AE	485 (42.1)	33 (39.8)	1		1	
CRF07_BC	356 (30.9)	14 (16.9)	0.52 (0.27–1.00)	0.050	0.57 (0.29–1.14)	0.113
CRF08_BC	183 (15.9)	25 (30.1)	2.19 (1.26–3.78)	0.005	2.03 (1.13–3.64)	0.018
CRF55_01B	79 (6.9)	3 (3.6)	0.53 (0.16–1.78)	0.308	0.57 (0.17–1.97)	0.405
Other^b^	48 (4.2)	8 (9.6)	2.74 (1.19–6.33)	0.018	2.73 (1.14–6.51)	0.024
**Year of diagnosis**						
2016	348 (30.2)	30 (36.1)	1			
2017	422 (36.7)	32 (38.6)	0.9 (0.54–1.52)	0.701		
2018	381 (33.1)	21 (25.3)	0.64 (0.36–1.15)	0.135		

^a^Adjusted for age, marital status, educational level, infection route, and subtype

^b^Other includes B subtype (*N* = 17, transmitted drug resistance = 7) and other subtype (*N* = 31, transmitted drug resistance = 1)

*DR* drug resistance, *OR* odds ratio, *CI* confidence interval, *MSM* men who have sex with men, *CRF* circulating recombinant form

### Analysis of HIV Drug Resistance Mutation Sites (DRMs) with subtype

**Fig. 2:**
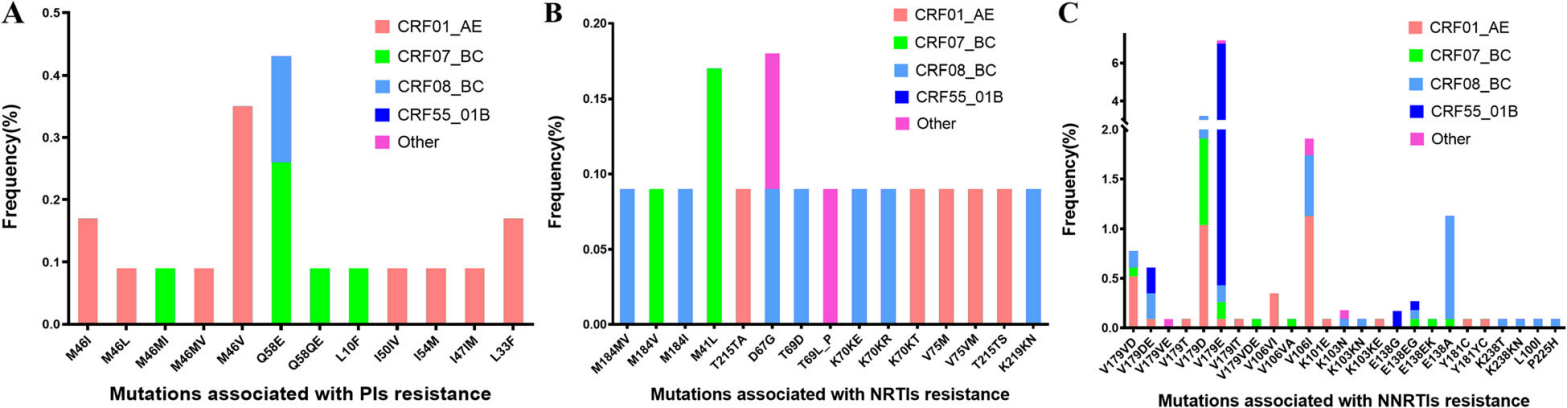
Analysis of drug resistance mutation sites with subtype

We identified 52 kinds of DRMs among the drug-resistant strains; 25 were associated with resistance to NNRTIs, 15 to NRTIs, and 12 to PIs. Individuals infected with the subtype CRF01_AE were the most likely to develop a PI-associated mutation, followed by those infected with CRF07_BC; the mutation sites primarily comprised M46V (0.35%) and Q58E (0.43%) mutations. Patients infected with the CRF01_AE subtype were the most likely to develop NRTI-associated mutations; the most common mutations were M41L (0.17%) and D67G (0.17%). Individuals infected with the subtypes CRF01_AE and CRF08_BC were the most likely to develop NNTRI-associated mutations; the most common mutations were V179E (7.21%), V179D (3.21%), and V106I (1.91%). The genotype CRF55_01B was the most likely to harbour V179E mutations (Fig. 2).

### Level of resistance to different antiretroviral drugs

The PI-associated mutations were predicted to confer resistance to atazanavir (0.09%), darunavir (0.17%), fosamprenavir (0.26%), indinavir (0.09%), Kaletra (also known as lopinavir/ritonavir; 0.17%), nelfinavir (1.13%), saquinavir (0.14%), and tipranavir (0.61%). The NRTI-associated mutations were predicted to confer resistance to abacavir (0.52%), zidovudine (also known as azidothymidine; 0.52%), stavudine (0.78%), didanosine (0.43%), emtricitabine (0.35%), lamivudine (0.35%), and tenofovir (0.26%). The NNRTI-associated mutations were predicted to confer resistance to doravirine (2.87%), efavirenz (1.04%), etravirine (0.87%), nevirapine (1.22%), and rilpivirine (2.17%). The drug resistance level was mostly categorised as low (Table 2).

**Table 2:** Analysis of resistance level against antiretroviral drugs

Drug	Drug resistance level				
	P N (%)	L N (%)	I N (%)	H N (%)	TDR N (%)
**PIs**					
ATV	10 (0.87)	1 (0.09)	0 (0.00)	0 (0.00)	1 (0.09)
DRV	0 (0.00)	2 (0.17)	0 (0.00)	0 (0.00)	2 (0.17)
FPV	12 (1.04)	0 (0.00)	1 (0.09)	2 (0.17)	3 (0.26)
IDV	11 (0.96)	1 (0.09)	0 (0.00)	0 (0.00)	1 (0.09)
LPV	4 (0.35)	1 (0.09)	1 (0.09)	0 (0.00)	2 (0.17)
NFV	8 (0.70)	10 (0.87)	3 (0.26)	0 (0.00)	13 (1.13)
SQV	4 (0.35)	2 (0.17)	0 (0.00)	0 (0.00)	2 (0.17)
TPV	3 (0.26)	7 (0.61)	0 (0.00)	0 (0.00)	7 (0.61)
**NRTIs**					
ABC	0 (0.00)	5 (0.43)	0 (0.00)	1 (0.09)	6 (0.52)
AZT	3 (0.26)	4 0.35)	1 (0.09)	1 (0.09)	6 (0.52)
D4T	1 (0.09)	7 (0.61)	2 (0.17)	0 (0.00)	9 (0.78)
DDI	6 (0.52)	4 (0.35)	1 (0.09)	0 (0.00)	5 (0.43)
FTC	2 (0.17)	0 (0.00)	1 (0.09)	3 (0.26)	4 (0.35)
3TC	2 (0.17)	0 (0.00)	1 (0.09)	3 (0.26)	4 (0.35)
TDF	0 (0.00)	2 (0.17)	0 (0.00)	1 (0.09)	3 (0.26)
**NNRTIs**					
DOR	1 (0.09)	31 (2.69)	0 (0.00)	2 (0.17)	33 (2.87)
EFV	137 (11.90)	5 (0.43)	4 (0.35)	3 (0.26)	12 (1.04)
ETR	173 (15.03)	6 (0.52)	4 (0.35)	0 (0.00)	10 (0.87)
NVP	162 (14.07)	6 (0.52)	2 (0.17)	6 (0.52)	14 (1.22)
RPV	158 (13.73)	19 (1.65)	5 (0.43)	1 (0.09)	25 (2.17)

*P* potential resistance, *L* low resistance, *I* intermediate resistance, *H* high resistance, *ATV* atazanavir, *DRV* darunavir, *FPV* fosamprenavir, *IDV* indinavir, *LPV* Kaletra, *NFV* nelfinavir, *SQV* saquinavir, *TPV* tipranavir, *ABC* abacavir, *AZT* zidovudine, *D4T* stavudine, *DDI* didanosine, *FTC* emtricitabine, *3TC* lamivudine, *TDF* tenofovir, *DOR* doravirine, *EFV* efavirenz, *ETR* etravirine, *NVP* nevirapine, *RPV* rilpivirine. Drug resistance = L + I + H

### Drug resistance-associated transmission cluster analysis

**Fig. 3:**
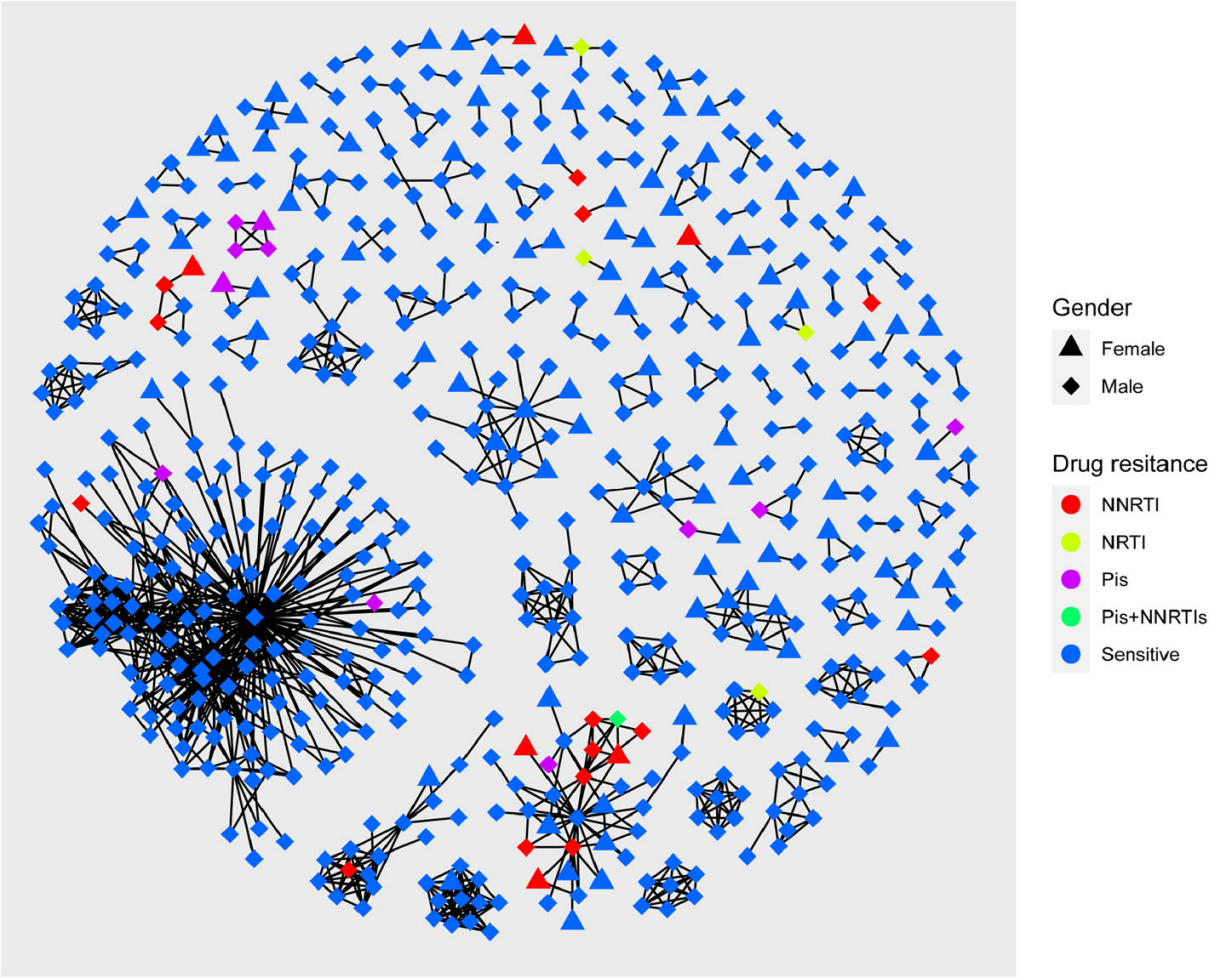
Drug resistance within human immunodeficiency virus 1 transmission clusters. PI, protease inhibitor; NRTI, nucleoside reverse transcriptase inhibitor; NNRTI, non-nucleoside reverse transcriptase inhibitor; sensitive, sensitive to antiretroviral drugs

We constructed an HIV-1 transmission network (Fig. 3). Of the 1151 subjects evaluated, 585 (50.8%) were segregated into 119 clusters, which included 490 men and 95 women. In the network, the cluster sizes ranged between 2 and 205; there were 96 (80.7%) clusters with size < 5, 14 (11.8%) clusters with 5 ≤ size < 10, and 9 (7.5%) clusters with size ≥10. The biggest cluster had 205 individuals (35.0%). (Fig. 3). We analysed the infection routes and found that 56.9% of the transmission cases occurred in heterosexual individuals, 40.7% in MSM, and 2.4% were of the IDU and MTCT. We also observed that 43.4% of TDR cases were included in the transmission network, and most (21, 58.3%) of which were primarily concentrated in four clusters. These TDR included 11 cases with resistance to PIs, 4 to NRTIs and 21 to NNRTIs, with the predominant gender being male (77.8%) and the major infection route being heterosexual transmission (83.3%). There were three clusters in which the TDR cases were connected with each other. We observed that there were four TDR females connected with 12 TDR males. To explore the factors associated with TDR, we performed bivariate and multivariate analyses. There was significant difference observed between the risk of TDR and unmarried status (aOR:4.24; 95%CI:1.51–11.9; *P*:0.006), level of junior high school or below (aOR:5.89; 95%CI:1.37–24.97; *P*:0.017), or infection with the CRF08_BC subtype (aOR:3.69; 95%CI:1.5–9.04; *P*:0.004) (Table 3).

**Table 3:** Factors associated with drug resistance transmission within clusters

Variables	N	Persons in TC	DR in TC	Crude OR (95% CI)	***p***	Adjusted OR (95% CI)^**a**^	***p***
**Age**							
< 35 years	508 (44.14)	288 (56.7)	7 (1.4)	1		1	
≥ 35 years	643 (55.86)	297 (46.2)	29 (4.5)	4.34 (1.87–10.08)	0.001	3.02 (0.94–9.73)	0.064
**Marital status**							
Married	479 (41.62)	221 (46.1)	17 (3.5)	1		1	
Unmarried	534 (46.39)	305 (57.1)	13 (2.4)	0.57 (0.28–1.19)	0.133	4.24 (1.51–11.9)	0.006
Divorced/widowed	126 (10.95)	50 (39.7)	3 (2.4)	0.77 (0.22–2.72)	0.680		
Unknown	12 (1.04)	9 (75.0)	2 (16.7)	6.0 (1.02–35.16)	0.047	6.26 (0.8–49.3)	0.081
**Educational level**							
College and above	336 (29.19)	203 (60.4)	3 (0.9)	1		1	
High school/ technical school	225 (19.55)	118 (52.4)	2 (0.9)	0.16 (0.04–0.69)	0.88	1.29 (0.2–8.39)	0.793
Junior high school/ below	590 (51.3)	264 (44.7)	31 (5.3)	8.87 (2.67–29.45)	0.000	5.89 (1.37–24.97)	0.017
**Ethnicity**							
Han	601 (52.22)	311 (51.7)	16 (2.7)	1			
Zhuang	483 (41.96)	235 (48.7)	16 (3.3)	1.35 (0.66–2.76)	0.409		
Other	68 (5.91)	38 (55.9)	4 (5.9)	2.18 (0.69--6.89)	0.186		
**Infection route**							
Heterosexual	693 (60.21)	333 (48.1)	32 (4.6)	1		1	
MSM	405 (35.19)	238 (58.8)	4 (1.0)	6.22 (2.17–17.83)	0.001	1.89 (0.51–6.99)	0.339
Other	53 (4.60)	14 (26.4)	0 (0.0)	–			
**Sex**							
Male	919 (79.84)	490 (53.3)	28 (3.0)	1			
Female	232 (20.16)	95 (40.9)	8 (3.4)	1.52 (0.67–3.44)	0.318		
**Subtype**							
CRF01_AE	485 (42.14)	226 (46.6)	12 (2.5)	1		1	
CRF07_BC	356 (30.93)	218 (61.2)	6 (1.7)	0.51 (0.19–1.37)	0.179		
CRF08_BC	183 (15.90)	81 (44.3)	17 (9.3)	4.74 (2.15–10.44)	0.000	3.69 (1.5–9.04)	0.004
CRF55_01B	79 (6.86)	47 (59.5)	0 (0.0)	–			
Other	48 (4.17)	13 (27.1)	1 (2.1)	1.49 (0.18–12.4)	0.714		
**Year of Diagnosis**							
2016	348 (30.23)	168 (48.3)	12 (3.4)	1			
2017	422 (36.66)	232 (55.0)	16 (3.8)	0.96 (0.44–2.09)	0.924		
2018	381 (33.1)	185 (48.6)	8 (2.1)	0.59 (0.23–1.48)	0.257		

^a^Adjusted for age, marital status, educational level, subtype

*TC* transmission clusters, *DR* drug resistance, *OR* odds ratio, *CI* confidence interval, *MSM* men who have sex with men, *CRF* circulating recombinant form

## Discussion

We aimed to reveal the genetic characteristics and prevalence of transmitted HIV TDR among the newly diagnosed individuals in Guangxi, China. Our study revealed that the major epidemic HIV-1 subtypes detected in Guagnxi were CRF01_AE (42.14%), CRF07_BC (30.93%), CRF08_BC (15.9%) and CRF55_01B (6.86%). The distribution of HIV-1 subtypes has changed in the last two decades. Subtypes B and E were the major prevalent strains before 2000 [2]. In 2013, Liu et al. found that CRF01_AE (77.6%), CRF08_BC (10.7%), and CRF07_BC (7.4%) were the major strains in Guangxi [21]. In 2015, Zhang et al. observed that CRF01_AE (62.0%), CRF07_BC (25.0%), and CRF08_BC (6.5%) were the major strains [22]. However, the proportion of individuals infected with CRF01_AE gradually declined, whereas the proportion of individuals infected with CRF07_BC increased [21, 22]. CRF07_BC was first detected in intravenous-drug users in Guangxi in 2002 [23]. To our knowledge, in this study, we observed the first incidence of CRF68_01B, CRF85_BC, and unconfirmed unique recombinant strains in Guangxi. Their routes of transmission warrant further study. These findings indicated the existence of high genetic heterogeneity and subtype/CRF diversity in HIV-1 in Guangxi, and showed that the new CRFs had spread to provinces with population movement. Furthermore, the continuation of HIV recombination led to the production of new CRFs and URF, which made the HIV subtypes more diverse and complex. Additionally, the introduction of the new CRFs had a profound impact on the local HIV epidemic, and made the spread across a floating population easier. Therefore, the surveillance of HIV subtype should be strengthened further.

According to the categorisation method established by WHO [24], the overall prevalence of TDR in Guangxi was at a moderate level (5–15%). It was higher than the prevalence determined in the region in a previous study [25] as well as in other regions of China [17, 26, 27]. As the increase in TDR will affect the antiviral treatment and the spread of drug resistance, the surveillance of TDR should be strengthened, and measures to curb the increase of TDR should be adopted. A significant difference was observed between the prevalence of TDR and marital status and subtype, which contradicted from the results of a previous study [17, 28]. When marital status was used as a categorisation parameter, unmarried individuals were most likely to develop drug resistance. When categorised based on subtype, patients with the CRF08_BC subtype were the most likely to develop drug resistance. Therefore, these two high-risk factors should be considered in clinical settings. We found a significantly higher prevalence of mutations related to NNRTI resistance than of those associated with resistance to PIs or NRTIs. Mutations related to NNRTI resistance were common, especially V179E (7.21%) and V179D (3.21%). Most V179E mutations were detected in CRF55_01B, and most V179D mutations were detected in CRF01_AE, CRF07_BC, and CRF08_BC subtypes. In case of NNRTIs, the mutation V106I, which can cause low-level resistance to doravirine, was a major cause of drug resistance. High-level resistance to efavirenz and nevirapine primarily resulted from the mutations K103N, L100I, and P225H. In case of NRTIs, 0.86% of the individuals were predicted to be resistant to zidovudine, stavudine, and didanosine. In case of PIs, 1.74% of the individuals were predicted to be resistant to nelfinavir and tipranavir. Combinatorial therapy with lamivudine, efavirenz, and nevirapine has been prescribed as the first-line ART regimen in China, and the total drug resistance rate to these three drugs found to be 2.61% in our study. Therefore, the emergence of these mutations might be related to the use of this first-line regimen. The higher prevalence of TDR may be caused by prolonged ART, as noted in other studies [29, 30].

To further understand the transmission of drug resistance, we constructed transmission clusters based on HIV-1 sequences. Our cluster analysis revealed that 585 individuals could be segregated into 119 clusters, including MSM, heterosexual men, heterosexual women, and intravenous-drug users; this indicated that the transmission characteristics were complicated. In the largest cluster, which comprised 146 individuals (1 woman, 145 men), MSM was the major route of transmission. Analysis of this super cluster suggested that MSM were strongly associated with the local epidemic. Additionally, 43.4% (36/83) of the individuals infected by drug-resistant strains were included in 19 clusters, in which drug resistance was mostly associated with heterosexuality (88.89%). Three clusters were identified that included drug-resistant individuals sharing a transmission relationship with each other, which counted for 44.4% (16/36). The result indicated that the spread of TDR may have occurred in the transmission network. The major infection route in the three clusters was heterosexual transmission (83.3%), and there were five women carrying TDR included in the TDR transmission network. This shows that drug-resistant individuals, especially women should be studied further, because they may act as a potential source of TDR. Logistic regression analysis revealed that being unmarried, having educational level of junior high school or lower, and subtype CRF08_BC may be related to TDR to transmit in the clusters. The reasons for the association of these parameters with TDR to transmit should be investigated further.

## Conclusions

The present study revealed a diverse, complex distribution of HIV-1 subtypes in Guangxi, and a moderate prevalence of TDR. However, there are several limitations in the study including the number of intravenous-drug users. Additionally, 13% of the samples could not be sequenced successfully, the major causes may include sample quality, low viral load in patients, or low sensitivity of reagents. The lack of data on contact between study participants was also another limitation. Future studies conducted on a larger, more diverse group with contact information available is necessary to design effective strategies for intervention in high-risk populations. Regardless, the risk of TDR was pronounced and must be factored into the treatment and prevention policies. Our findings will be helpful in determining the optimal primary ART and implementing effective interventions that target the major populations at risk in the area.

## Data Availability

The datasets are available from the corresponding author on reasonable request. Some of the sequences are accessible at GenBank (accession numbers, MW294209 - MW295352).

## Abbreviations

HIV: Human immunodeficiency virus
MSM: men who have sex with men
TDR: Transmission drug resistance
ART: Antiretroviral therapy
NNRTI: Non-nucleoside reverse-transcriptase inhibitor
NRTI: Nucleoside reverse-transcriptase inhibitor
PI: Protease inhibitor
EFV: Efavirenz
NVP: Nevirapine
FTC: Emtricitabine
3TC: Lamivudine
ABC: Abacavir
DDI: Didanosine
D4T: Stavudine
TDF: Tenofovir
AZT: Azidothymidine
DRV/r: Darunavir
LPV/r: Lopinavir
ATV/r: Atazanavir
DRM: Drug resistance mutation
HIVDR: HIV Drug Resistance Database
TN93: Tamura-Nei 93
OR: Odds ratio
aOR: Adjusted odds ratio
CI: Confidence interval
